# Supplementary material for: Radiomic and Volumetric Measurements as Clinical Trial Endpoints—A Comprehensive Review
Source: Cancers (Basel). 2022 Oct 17;14(20):5076. doi: 10.3390/cancers14205076 (PMC9599928; doi:10.3390/cancers14205076)

cancers

MDPI

Supplementary Materials

Supplementary Section S1: Search strategy

PubMed search strategy: Title and/or abstract including: ((cancer[Title/Abstract]) OR tumor[Title/Abstract]) OR (tumor[Title/Abstract]) OR (oncology[Title/Abstract]) OR (oncological[Title/Abstract]) OR (malignant[Title/Abstract]) OR (malignancy[Title/Abstract])) AND ((radiomic[Title/Abstract]) OR (radiogenomic[Title/Abstract]) OR (volumetric[Title/Abstract]) OR (volume[Title/Abstract])) AND (computational[Title/Abstract]) OR (comput[Title/Abstract]) OR (model[Title/Abstract]) OR (machine[Title/Abstract]) OR (machine learning [Title/Abstract]) OR (deep learning[Title/Abstract]) OR (framework[Title/Abstract])) AND ((predict[Title/Abstract]) OR (prediction[Title/Abstract]) OR (predicts[Title/Abstract])) AND ((response[Title/Abstract]) OR (respond[Title/Abstract]) OR (RECIST[Title/Abstract]) OR (pathological[Title/Abstract]) OR (pathol*[Title/Abstract]) OR (pathological response[Title/Abstract]) OR (survival[Title/Abstract]) OR (surviv*[Title/Abstract]) OR (outcome[Title/Abstract]) OR (outcomes[Title/Abstract]) OR (PFS[Title/Abstract])).

Supplementary Table S1: Study selection: inclusion/exclusion criteria

	Include	Exclude
Language	English	Non-English
Article type	Primary research papers only	Reviews<br>Systematic reviews<br>Opinion pieces
Study type	Observational studies (retrospective or prospective)<br>Interventional (retrospective or prospective)	Studies NOT involving human patients<br>Studies NOT involving radiological imaging of human patients
Population	Adults aged 18 or above<br>Solid tumors<br>Undergoing oncological treatment (any oncological treatments, i.e. systemic/ RT/surgical/other)	Non-malignant conditions<br>Age <18
'Intervention' or 'Test' – in our case the imaging-based predictive model	Radiomic or radiogenomic models, or<br>Models using tumor volumetric data based on CT/MRI/PET/US images<br>Type of model: machine learning model, or other statistical models.	Studies NOT involving radiomics or volumetric analysis<br>Studies NOT evaluating prediction of treatment response or outcome<br>Technical studies that use ML to improve e.g. segmentation accuracy, but do not aim to evaluate prediction of treatment response or outcome<br>Methodologies that do NOT allow prediction based on pre-treatment imaging alone (e.g. by using a combination of pre- and post-treatment imaging)
Comparison	RECIST – optional (but to be noted)?<br>Integration with clinical data – optional (but to be noted)?	
'Outcome' – in our case outcome/success of predictive model	Predicting response (decision re measures to include – any response markers, or only validated surrogate endpoints – pending further search)<br>Predicting PFS/OS	Any papers that only predict OS, but no measures that relate specifically to the given line of treatment (e.g. path CR, PFS).

Supplementary Section S2: Quality assessment method

Patient characteristics
Histological subtype(s)
Any molecular selection criteria – e.g. specific mutations?
Any other specific inclusion/exclusion criteria of note
Line of treatment (e.g. first line or at recurrence etc)
Study design and data set
Retrospective vs prospective?
Sample size (n =)
Were sample size calculations done/included?
Single or multi-institution?
Internal validation? Yes/No
External validation? Yes/No
Imaging information
Modality (i.e. CT, MRI, PET, etc)
Comment on imaging analysis method (number of assessors/segmenting Radiologists)
Other data types
Clinical data included? Yes/No – If yes, what data types were included
Molecular data included? Yes/No – If yes, what was included
Algorithm
Type of algorithm
Algorithm made available?
What was predicted by the model? E.g. PFS, TTP, OS, pathological CR, etc
Model performance
What was model performance benchmarked against?
Raw data reported e.g. TN/FN/TP/FP contingency table?
Accuracy metrics reported? (include confidence intervals, p-values etc if reported. E.g.

- AUC/AUROC?
- *F* score or *F1* score?
- Accuracy?
- Sensitivity, specificity?
- PPV/NPV?

Comment on generalisability/reproducibility
Has the model received regulatory approval, e.g. FDA approval? Yes/No

Supplementary Figure S1: Timeline of reviewed publications

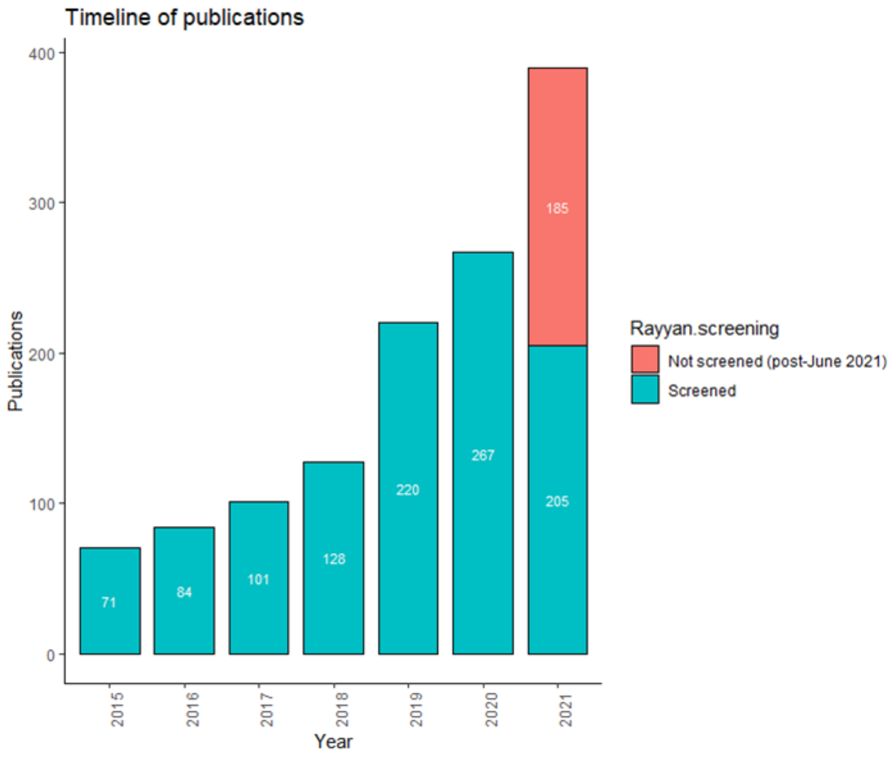

Supplement: Supplementary file 1 [file cancers-14-05076-s001.zip › cancers-1943666-supplementary.pdf]
